# Yeast Based Small Molecule Screen for Inhibitors of SARS-CoV

**DOI:** 10.1371/journal.pone.0028479

**Published:** 2011-12-02

**Authors:** Matthew Frieman, Dipanwita Basu, Krystal Matthews, Justin Taylor, Grant Jones, Raymond Pickles, Ralph Baric, Daniel A. Engel

**Affiliations:** 1 Department of Microbiology and Immunology, University of Maryland, Baltimore, Maryland, United States of America; 2 Department of Microbiology, University of Virginia School of Medicine, Charlottesville, Virginia, United States of America; 3 Department of Microbiology and Immunology, Gene Therapy Center, University of North Carolina, Chapel Hill, North Carolina, United States of America; 4 School of Public Health, University of North Carolina, Chapel Hill, North Carolina, United States of America; Kantonal Hospital St. Gallen, Switzerland

## Abstract

Severe acute respiratory coronavirus (SARS-CoV) emerged in 2002, resulting in roughly 8000 cases worldwide and 10% mortality. The animal reservoirs for SARS-CoV precursors still exist and the likelihood of future outbreaks in the human population is high. The SARS-CoV papain-like protease (PLP) is an attractive target for pharmaceutical development because it is essential for virus replication and is conserved among human coronaviruses. A yeast-based assay was established for PLP activity that relies on the ability of PLP to induce a pronounced slow-growth phenotype when expressed in *S. cerevisiae*. Induction of the slow-growth phenotype was shown to take place over a 60-hour time course, providing the basis for conducting a screen for small molecules that restore growth by inhibiting the function of PLP. Five chemical suppressors of the slow-growth phenotype were identified from the 2000 member NIH Diversity Set library. One of these, NSC158362, potently inhibited SARS-CoV replication in cell culture without toxic effects on cells, and it specifically inhibited SARS-CoV replication but not influenza virus replication. The effect of NSC158362 on PLP protease, deubiquitinase and anti-interferon activities was investigated but the compound did not alter these activities. Another suppressor, NSC158011, demonstrated the ability to inhibit PLP protease activity in a cell-based assay. The identification of these inhibitors demonstrated a strong functional connection between the PLP-based yeast assay, the inhibitory compounds, and SARS-CoV biology. Furthermore the data with NSC158362 suggest a novel mechanism for inhibition of SARS-CoV replication that may involve an unknown activity of PLP, or alternatively a direct effect on a cellular target that modifies or bypasses PLP function in yeast and mammalian cells.

## Introduction

Highly pathogenic respiratory viruses, like the H5N1 influenza virus and severe acute respiratory syndrome coronavirus (SARS-CoV), represent significant threats to public health and global economic stability. They cause acute lung injury (ALI) that rapidly progresses to acute respiratory distress syndrome (ARDS), the former most notably in the elderly [1], [2], [3]. Moreover, after viral clearance many SARS and H5N1 patients develop diffuse alveolar damage (DAD) that often progresses to pulmonary fibrosis, another devastating end stage lung disease, characterized by dysregulated cell proliferation during wound repair[4], [5], [6].

SARS first emerged in China in 2002, the result of SARS-CoV crossing the species barrier from bats followed by amplification and additional mutations occurring in other species such as civet cats and raccoon dogs, which allowed for transmission to humans [7], [8]. In many cases infection resulted in severe acute respiratory disease, pneumonia and death [9], [10]. Over 8000 cases and ∼800 deaths were reported worldwide between 2002 and 2004 and many patients required mechanical ventilation and intensive care [11], [12]. In late 2003 and early 2004, newly infected persons were identified with SARS-CoV strains such as GDO3, which was significantly different from those predominating in the 2002-2003 outbreaks [13]. These events indicate that a SARS epidemic may recur, emerging from SARS-CoV strains circulating in bats, civets or raccoon dogs.

The papain like protease (PLP) is an essential component of the SARS-CoV replication machinery. PLP is a domain of the nsp3 protein that is initially synthesized as the ORF1a polyprotein during replication, which then cleaves protease recognition sites between nsp1/2, nsp2/3 and nsp3/4 [14]. In addition to protease activity PLP has deubiquitination, and interferon antagonist activities *in vitro* [*15*]. Homologues of PLP are found in all coronaviruses so its targeting for drug discovery is likely to be important for both SARS-CoV and other human coronaviruses.

We have developed a yeast-based assay and screening method to identify small molecules that block SARS-CoV replication based on their inhibition of PLP. The basis for the screen is that forced expression of PLP in *S. cerevisiae* causes a pronounced slow growth phenotype. Using this finding we screened a small molecule library for compounds that specifically reversed the PLP-induced slow growth phenotype. These compounds were then tested in cell culture models for efficacy against SARS-CoV replication, as well as the known enzymatic functions of PLP. Here we report that of 5 compounds that reversed the slow growth phenotype in yeast; 1 compound, NSC158362, also significantly blocked SARS-CoV replication *in vitro* with an EC50 <1 µM. This effect was specific for SARS-CoV replication because no effect on influenza virus replication was observed with up to 50 µM of the inhibitory compound. A second compound, NSC158011, was able to inhibit PLP-dependent protease activity in a cell culture assay but this effect did not appear strong enough to block virus replication. Interestingly, NSC158362 failed to block the protease, deubiquitinase or anti-IFN activities of PLP. This suggests that its target is either a novel activity of PLP or is a cellular protein that regulates PLP function in infected cells, thus representing new avenues of therapeutic intervention for SARS-CoV.

## Results

### PLP expression slows *S. cerevisiae* cell growth

**Figure 1:**
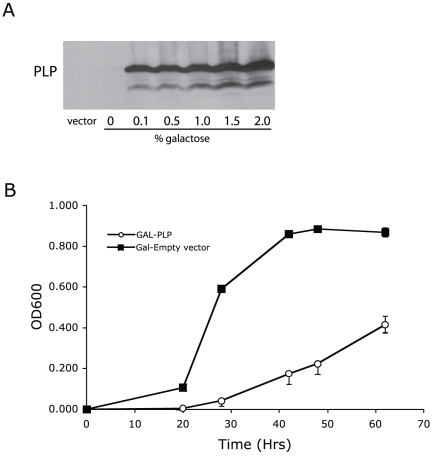
SARS-CoV PLP produces a slow growth phenotype in yeast. A. Galactose induction of SARS-CoV PLP. Strain containing HA tagged PLP under the control of the yeast *GAL1* promoter was grown in the presence of 0 to 2% galactose. Protein was extracted and analyzed by anti-HA western blot. B. Growth curve of yeast expressing either empty vector or HA tagged PLP grown in 2% galactose media.

Previously we reported that expression of the influenza virus NS1 protein in yeast resulted in a slow growth phenotype that could be used to screen for specific small molecule antagonists of NS1 [16]. We reasoned that expression of SARS-CoV proteins in yeast may modulate cellular processes including signaling pathways, as they do in mammalian cells, allowing for a genetic system to analyze their function and the capability to identify compounds that alter that function. We focused on the PLP protease domain of the viral nsp3 protein because of its requirement in virus infection [17]. Sequences corresponding to the PLP domain were cloned into a plasmid containing the galactose-inducible *GAL1* promoter for controlled expression in *S. cerevisiae*. The plasmid was transformed into a modified strain of *S. cerevisiae* that carries disrupted alleles for two genes that control drug efflux, *PDR1* and *PDR3*, thus allowing for the efficient retention of small molecules [16]. Shown in Figure 1A is a galactose induction experiment of the PLP expressing strain demonstrating increasing induction with increasing galactose concentration. High-level expression of PLP was observed with as little as 0.1% galactose, and 2% galactose was chosen for further studies. We next analyzed the effect of PLP expression on yeast growth in liquid media. Growth in medium containing glucose, which represses *GAL1*-driven expression, resulted in both the control and PLP strains growing equally well, as expected (data not shown). Growth in galactose-containing medium was performed over a 60 hour time course and the results are shown in Figure 1B. There was a clear growth inhibition due to expression of PLP, with the maximal differential between control and PLP strains occurring between 30 and 50 hours after initiating growth in galactose-containing medium.

### Screen for chemical suppressors of the PLP-induced slow-growth phenotype

**Figure 2:**
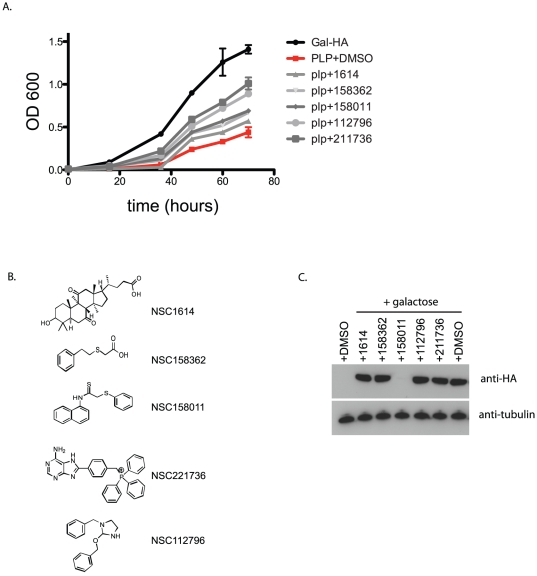
Compounds that reverse the slow growth phenotype. Yeast grown in media containing 2% galactose with the addition of either 1% DMSO or 50 uM compounds dissolved in 1% DMSO. B. Structures of compounds shown in A. C. Effects of compounds on PLP expression. Western blots were performed with protein extracted from HA tagged PLP expressing yeast grown in the presence of 2% galactose and 50 uM of each compound and visualized with anti-HA antibody.

Cells from an overnight culture were plated in a 96-well format at 5×10^5^ cells/ml in 100 µl of galactose-containing medium in the presence of 50 µM of each test compound or 1% DMSO as control. Approximately 2,000 compounds from the NIH Developmental Therapeutics Program (DTP) Diversity Set library (http://dtp.nci.nih.gov/index.html) were screened manually. Cell growth was monitored by optical density (OD) over the course of 60 hours. Hits were identified as those producing a 1.3 fold or greater increase in OD compared to the DMSO control. Compounds positively affecting yeast growth were tested for reproducibility using independent samples of each compound obtained from the DTP. Five compounds demonstrated reproducible activity and their effects on growth of the PLP strain are shown in Figure 2A. The structures of the five compounds are presented in Figure 2B.

### Effects on PLP expression

One explanation for the restoration of yeast growth could be a reduction in PLP protein levels. This was examined by western blot analysis using the C terminal HA tag that was fused to PLP in the expression construct. Cells containing the PLP plasmid were induced with 2% galactose for 18 hours in the presence of 50 µM of each compound. As shown in Figure 2C, expression of PLP was unaffected by four of the five compounds, however NSC158011 triggered a significant decrease in PLP expression. These data indicate that in yeast, with the exception of NSC158011, hits from the screen acted either directly at the level of PLP function to suppress the slow-growth phenotype, or alternatively they acted on cellular processes that specifically modify or bypass PLP function without altering its expression.

### Toxicity studies

**Figure 3:**
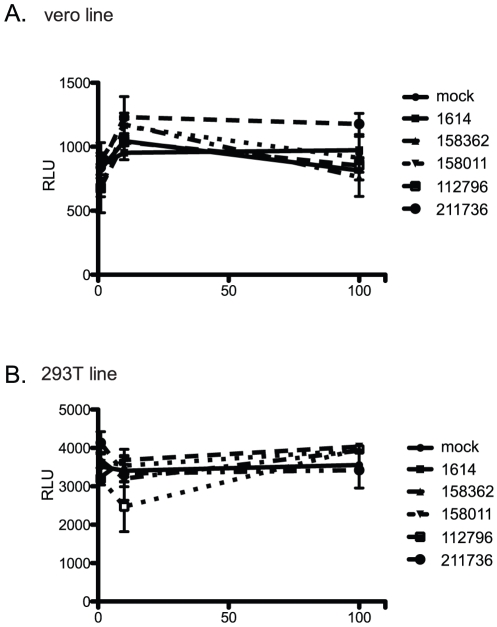
Toxicity assays. VeroE6 (A) or 293T (B) cells were treated with 0, 10 uM or 100 uM of each compound in 1% DMSO for 24 hours. Cells were analyzed for viability with the CellTiter Glo assay (Promega).

Each compound was tested for toxicity in both 293T and VeroE6 cells to confirm the levels used in antiviral assays were safe for cells. Cells were treated with each of the 5 compounds at 1, 50 and 100 µM concentrations for 24 hours, after which cell viability was analyzed by the CellTiter-glo viability assay (Promega). We found no toxicity of any of the five compounds even at 100 µM concentration in either of the cell lines (Figure 3A and B).

### Effects on SARS-CoV replication in vitro

**Figure 4:**
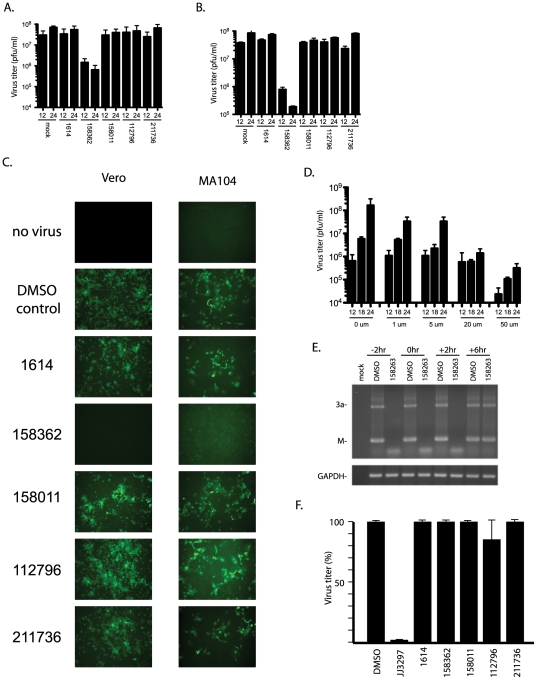
Effects of compounds on virus growth and RNA production. VeroE6 (A) or MA104 (B) cells were treated with 50 uM of each compound or 1% DMSO alone and infected with SARS-CoV(GFP) at an MOI of 3. Virus titer was assayed by plaque assay on VeroE6 cells after 12 and 24 hours of growth. C. Fluorescence images of SARS-CoV(GFP) infected Vero and MA104 cells at 24 hours post infection. D. Dose curve of NSC158362 on SARS-CoV growth. Various concentrations of drug were added to Vero cells 2 hours before SARS-CoV was added at an MOI of 3. Aliquots were removed at 12, 18 and 24 hours post infection and titered on Vero cells. E. Vero cells were treated with either DMSO or NSC 158263 at -2 hours, 0 hours, + 2 hours or +6 hours after infection with SARS-CoV. RNA was isolated at 12 hours post infection and analyzed by RT-PCR for SARS-CoV specific transcripts and GAPDH. F. MDCK cells were treated with 50 uM of each compound or 1% DMSO alone and infected with influenza A/PR/8 at an MOI of 0.1. Virus titer was determined by hemagglutination assay.

The effect of each compound on SARS-CoV replication was tested in Vero E6 and MA104 cells. Initially, cells were plated in 24 well plates and treated with 50 µM of each compound for 2 hours prior to infection (Figure 4). At 2 hours after treatment, the medium was removed and the cells infected with a GFP expressing version of SARS-CoV at an MOI of 1. After 1 hour, the cells were washed twice with PBS and then medium containing each compound, at the original concentration, was added. At 12 and 24 hours post infection aliquots were analyzed by plaque assay on Vero E6 cells (Figure 4A and B) and by fluorescence microscopy (Figure 4C). In control, DMSO treated cells, SARS-CoV grew to ∼5X10∧7 by 12 hours post infection and ∼1×10∧8 by 24 hours post infection. Of the five compounds tested, NSC158362 reduced the viral titer by 100 fold at 12 hours post infection and greater than 500 fold at 24 hours post infection (Figure 4A,B). At 12 hours post infection strong fluorescence was seen for DMSO-treated cells and those treated with NSC1614, NSC158011, NSC112796 and NSC211736. Additionally, we observed a strong cytopathic effect (CPE) in these cells as well as evidenced by rounding up, cell fusion and ruffling of the plasma membrane (not shown). However in cells treated with NSC158362 minimal GFP expression and CPE was observed. Therefore by two independent measurements, plaque assay and GFP expression; our data indicate that NSC158362 exhibited antiviral activity against SARS-CoV in cell culture.

We next performed a dose response experiment with NSC158362. Cells were treated with increasing concentrations of the compound starting at 2 hours prior to infection and continuing through 24 hours post-infection (Figure 4D). The cell supernatants were analyzed for virus replication at 12, 18 and 24 hours post infection. We observed a clear dose-dependent effect of NSC158362. For all concentrations tested the greatest effects occurred when the infection and treatment were carried out for 24 hours. Under these conditions the EC50 was less than 1 uM. The maximal effect on replication was greater than 500 fold at a concentration of 50 uM. These data demonstrate the strong inhibitory effects of NCS158362 on SARS-CoV replication.

Additionally, we examined the effect of NSC158362 on viral subgenomic RNA (Figure 4E). Vero cells were infected at an MOI of 3 either 2 hours after NSC158362 was added, at the time of NSC158362 addition, 2 before NSC158362 was added or 6 hours before NSC158362 was added. SARS-CoV was incubated until 12 hours post infection, at which time RNA was extracted and viral replication was analyzed by RT-PCR using primers specific for subgenomic transcripts. We find that the addition of NSC158362 either prior to infection, at the time of infection or 2 hours after infection can very efficiently inhibit virus RNA production, but by 6 hours after virus addition, the anti-viral effects of NSC158362 are significantly reduced.

### Specificity of inhibition

We examined the specificity of the compounds for SARS-CoV growth inhibition by challenging the replication of influenza virus. MDCK cells were treated with each of the five compounds at a concentration of 50 uM and then infected with influenza virus A/PR/8 at an MOI of 0.1 for 48 hours (Figure 4F). As a positive control infected cells were also treated with compound JJ3297, which we have shown previously to dramatically inhibit influenza virus replication through inhibition of viral NS1 protein function[18]. We observed no reduction in influenza virus replication with NSC159362, confirming that their effect on SARS-CoV replication is specific.

### Efficacy of compounds against SARS-CoV in human airway epithelial cells

**Figure 5:**
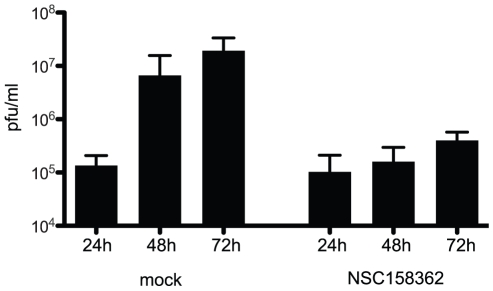
Inhibition of SARS-CoV replication in human airway epithelial cells by NSC158362. HAE cells were treated with either 1% DMSO or NSC158362 at 50 uM and infected with SARS-CoV(GFP). The apical surface of each culture was rinsed with PBS at 24, 48 and 72 hr post infection and virus titered on VeroE6 cells.

The analysis of efficacy in Vero E6 and MA104 cells is limited since they are not human cells and they do not reflect the complex architecture of the lung. We therefore used human airway epithelial cells (HAE) to further assess the *in vitro* efficacy of NSC158362 (Figure 5). HAEs are derived from primary human airway cells and are grown on trans-wells that result in an air-liquid interface where the apical surface of the well contains beating cilia, clara cells and goblet cells with very little media remaining on the surface[19]. The basolateral surface is bathed in growth media similar to the architecture of lungs *in vivo*. HAEs were treated with either DMSO or NSC158362 on the apical and basolateral surface for 2 hours prior to infection. SARS-CoV/GFP was added to the apical surface of the transwell at an MOI of 3 for 1 hour in a volume of 100 µl. At 1 hour, the wells were washed twice with PBS and incubated for 3 days in the presence of NSC158362 at 50 µM. A previous study showed that SARS-CoV is only released from the apical surface of HAEs[20]. At 24, 48 and 72 hours post infection the apical surface was washed with 200 µl of PBS and that apical wash used to assay the growth of the virus by plaque assay on Vero E6 cells as above (Figure 5). SARS-CoV infection of DMSO-treated HAEs had titers that of 2×10∧7 pfu/ml at 72 hours post infection. However, SARS-CoV infected HAEs treated with NSC158362 had a titer of 4×10∧5 pfu/ml at 72 hours post infection, a >50 fold reduction in titer. This demonstrates that NSC158362 is effective at inhibiting SARS-CoV replication in a physiologically relevant cell type.

### Effects on PLP protease activity

PLP has protease, de-ubiquitinase and IFN antagonist activities. To explore the mechanism of NSC158362's antiviral activity we performed cell culture assays to determine if any of the known the enzymatic activities of PLP were inhibited.

Previously we developed a PLP cleavage assay that makes use of a plasmid expressing a fusion protein of nsp2/3/GFP as a reporter for PLP cleavage [21]. If PLP activity is retained then the nsp2/3/GFP fusion protein is cleaved into two polypeptides, one with just nsp2 and one with nsp3/GFP. Western blotting for GFP reveals a size shift from the full length nsp2/3/GFP to the smaller nsp3/GFP fragment.

**Figure 6:**
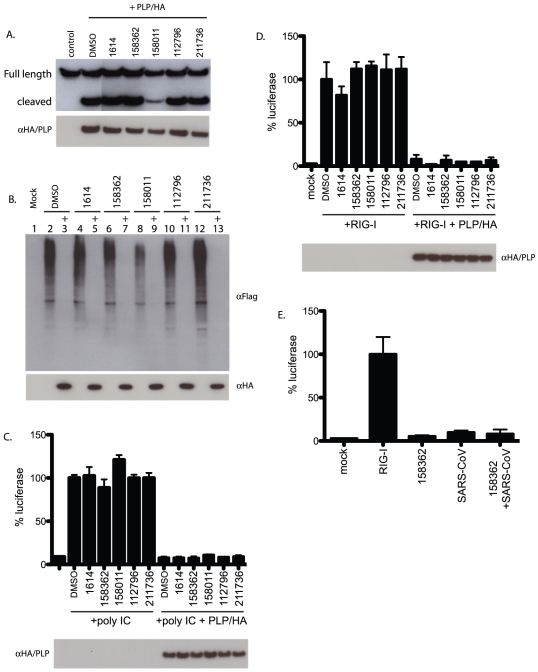
Effect of compounds on PLP enzymatic function. A. PLP protease activity was assayed using a nsp2/3/GFP reporter plasmid. 293T cells were transfected with either nsp2/3/GFP alone or with HA tagged PLP. Cells were treated with 50 uM of each compound or 1% DMSO alone and protease activity was assayed by reduction in size of the nsp2/3/GFP fusion protein by western blot with anti-GFP antibody. B. PLP deubiquitinase activity was assayed in cells transfected with Flag tagged ubiquitin and HA tagged PLP. 293T cells were transfected with both plasmids. Cells were treated with 50 uM of each compound or 1% DMSO alone and deubiquitinase activity was assayed by reduction in ubiquitinated protein by western blot with anti-Flag antibody. C and D. Effects of the compounds on PLP's IFN antagonism ability were analyzed by poly IC treatment of RIGI transfection of cells with and IFNβ/luciferase reporter plasmid with and without PLP and the compounds. Western blot of transfected HA/PLP shown below each graph. E. Effect of 158362 on IFN induction after infection with SARS-CoV. Vero cells were transfected with IFNβ/luciferase reporter plasmid. RIG-I was transfected in 1 set of wells as a positive control. Cells were then treated with either DMSO or 158362 for 2 hours prior to infection with SARS-CoV at an MOI of 3. No increase in IFNβ induction was seen after infection.

293T cells were co-transfected with a plasmid expressing PLP and a plasmid expressing the nsp2/3/GFP fusion protein. Four hours after transfection each compound under study was added to the wells at a concentration of 100 µM and allowed to incubate overnight. At 18 hours post transfection the cells were lysed and analyzed by SDS-PAGE and western blotted with anti-GFP antibody (Figure 6A). In control cells without PLP only the large uncleaved full-length protein was observed. In contrast, in cells expressing PLP a smaller cleaved protein was released at the expected molecular weight. In cells treated with each of the compounds only NSC158011 produced a decrease in the amount of the cleavage product. However, since NSC158011 had no effect on viral replication this suggests that the residual PLP protease activity in these cells was enough to fully support viral replication. Nonetheless this result demonstrates that one of the hits from the screen had an effect on the protease activity of PLP. In NSC158362 treated cells there was no effect on PLP-dependent protease function, suggesting that the inhibition of PLP protease function is not the mechanism of NSC158362's antiviral action.

### Lack of effect on PLP de-ubiquitinase activity

We next assayed for the ability of the compounds to inhibit the deubiquitinase (DUB) activity of PLP. We have previously shown that when PLP is co-transfected with a Flag-tagged ubiquitin plasmid into 293T cells, complete de-ubiquitination of the proteins in the cell is observed [21]. To assay the effect of the compounds on PLP DUB activity, plasmids expressing PLP and Flag tagged ubiquitin (Flag/Ub) were co-transfected into 293T cells. At 4 hours post transfection each compound was added at a concentration of 50 µM. The cells were incubated for 18 hours before protein was extracted and assayed for incorporation of Flag tagged ubiquitin by western blot analysis with an anti-Flag antibody. In Figure 6B, Flag/Ub is expressed alone in lane 2 and high molecular weight species corresponding to ubiquitinated proteins were found. In lane 3, where PLP is co-transfected with the Flag/Ub plasmid, a total lack of Flag-conjugated protein signal was seen, demonstrating the strong DUB activity of PLP. In lanes 4, 6, 8, 10 and 12 the cells were treated with each compound and transfected with only the Flag/Ub plasmid. These lanes show equally strong ubiquitination of cellular proteins. However in lanes 5,7,9,11 and 13 where PLP was co-transfected into the treated cells a total lack of ubiquitination signal was observed in each lane. This suggests that there was no inhibition of PLP's DUB activity by any of the compounds identified including NSC158362, which inhibited SARS-CoV replication.

### Lack of effect on PLP IFN antagonism

We have previously shown PLP to be a potent IFN antagonist *in vitro* [10]. Expression of PLP in cells blocks the IRF3 signaling pathway and inhibits the induction of IFNβ. Compounds that inhibit PLP's IFN antagonism would be expected to restore induction of IFNβ. We assayed for IFNβ induction by transfecting cells with an IFNβpromoter/luciferase reporter plasmid and treatment with poly IC to induce the IFNβpromoter. The cells were also transfected with our PLP-expressing plasmid (PLP/HA), in the presence or absence of compounds (Figure 6C). At 18 hours post transfection the cells were analyzed for luciferase induction. None of the compounds reversed the IFN antagonism activity of PLP/HA. We next co-transfected the reporter plasmid with a RIG-I expressing plasmid to induce IRF3, and also the PLP/HA plasmid. All three plasmids were co-transfected into 293T cells and 4 hours post transfection each compound was added at a concentration of 100 µM. At 18 hours post transfection the cells were analyzed for luciferase induction (Figure 6D). When PLP was not transfected with the RIG-I plasmid and the IFNβ/luciferase reporter plasmid, a robust induction of luciferase was produced signifying a strong induction of the IFNβ promoter. When PLP was co-transfected with the other two plasmids and treated with 1% DMSO as a control, a strong inhibition of IFNβ induction was observed. However, when cells were treated with each of the compounds PLP was still able to inhibit the induction of IFNβ expression. This indicates that none of the compounds blocked PLP's IFN antagonism activity.

We next tested the effect of the NSC158362 on SARS-CoV induction of IFNβ directly (Figure 6E). Vero cells were transfected with an IFNβ luciferase reporter plasmid for 18 hours. A control transfection was performed containing the luciferase reporter plasmid and a plasmid expressing RIG-I, a potent inducer of IFNβ. They were then treated with either DMSO or NSC158362 for 2 hours prior to an infection with SARS-CoV at an MOI of 3. At 8 hours post infection, cells were analyzed for their ability to induce IFNβ after viral infection. We find no increased IFNβ production after treatment with NSC158362 and infection with SARS-CoV suggesting the drug is not affecting the ability of SARS-CoV to inhibit the innate immune response.

## Discussion

Novel strategies to identify new antiviral compounds are needed. The 2009 H1N1 pandemic, the SARS-CoV epidemic and the emergence and spread of West Nile virus demonstrate that current antiviral therapies will not work for all new and emergent viruses. As the world's human population expands and interacts more and more with the environment, an increase in viral outbreaks is inevitable. We have developed a novel screen for antiviral compounds that is rapid, direct and does not rely on previous knowledge of a viral protein's function. The yeast based screen described here was used to identify an antiviral compound directed against the SARS-CoV papain-like protease. While the function of PLP in SARS-CoV replication largely understood, this was not necessary for the yeast-based screening methodology described here to be successful. Initially, several SARS-CoV proteins were tested in *S. cerevisiae* for their ability to inhibit yeast cell growth in an inducible manner (not shown). Once identified as strongly growth inhibited by PLP, yeast were then challenged with the 2000 member NIH Diversity Set for compounds that reversed the inhibition of yeast cell growth. Five compounds passed the screen and those were tested against SARS-CoV infection *in vitro*, of which 1 compound proved to be a potent antiviral.

We found that NSC158362 is able to block SARS-CoV replication by more than 500 fold in culture. We also showed that NSC158362 has a strong anti-SARS-CoV effect using HAE cells, a physiological model of lung architecture containing ciliated cells that are the *in vivo* target of the virus.

We do not know the precise mechanism of this compound's action. It was identified by the ability to reverse the PLP-induced slow growth phenotype in yeast. The compound could be functioning at many possible levels, including (1) blocking PLP:host protein interactions (2) inhibiting an unknown enzymatic activity of PLP or (3) inhibiting a cellular function that modifies PLP or regulates its function. It could also be acting at the cell surface in a way that triggers a modulation of the PLP-induced signaling pathway. Finally, it could be acting downstream of the effects of PLP in infected cells, so as to bypass the effects of PLP. Regardless it is clear that compound NSC158362 specifically inhibits SARS-CoV replication (but not influenza virus replication) as well as SARS-CoV RNA production in infected cells. Further investigation of the target of NSC158362 will likely yield novel insights into SARS-CoV replication and also provide new avenues for therapeutic intervention.

We examined the effect of these five hits on the known PLP enzymatic activities including protease function, de-ubiquitination and IFN antagonism. Interestingly, despite a lack of antiviral activity, compound NSC158011 diminished PLP-dependent protease activity in a cell culture assay (Figure 6A). Since the effect on protease activity was only partial, we conclude that the effect was not strong enough to lead to a diminution of virus replication. The precise effect of NSC158011 on protease activity could be due to several factors. These include (1) direct inhibition of the protease activity (2) inhibition of a cellular protein whose function is required for PLP activity in cells or (3) triggering the degradation of PLP by direct binding or other mechanisms. With the exception of NSC158011's effect on protease activity, our assays showed that none of the compounds had an effect on PLP's known enzymatic activities. We hypothesize that this compound is either affecting an unidentified activity of PLP or that it acts at the level of a cellular protein that modifies or bypasses the function of PLP in cells. Given that NSC158362 is functional not only in yeast but also in mammalian cells, it is very likely that the target of this compound is PLP itself or a cellular protein that is highly conserved from yeast to humans.

We have employed a novel antiviral screen to identify a compound that specifically inhibits SARS-CoV replication in multiple cell lines. Use of the yeast based screen to identify antivirals is rapid and efficient, both important aspects when dealing with newly emerging infectious diseases. Since knowledge of the function of the viral protein is not required in order to perform this type of small molecule screen, it can be scaled to any size virus and rapidly initiated once the viral sequence is known of a pathogen, potentially leading to the direct identification of lead compounds for further adaptation and testing *in vivo.*

## Methods

### Plasmids

A previously cloned version of PLP containing a HA tag [10] was used to PCR amplify PLP for cloning into the galactose inducible yeast expression vector pRS416, to create pRS416/PLP/HA. PLP/HA [10] under the control of chicken β-actin promoter (pCAGGS-PLP/HA) and a reporter plasmid encoding firefly luciferase under the control of IFN-β promoter (IFNβ/luciferase) were identical to those used in [10].

### Yeast strains and growth

Strain 9526-6-2 (*MAT*a *his3Δ1 leu2Δ0 lys2Δ0 ura3Δ0 pdr1::KanMX4 pdr3::KanMX4*) was a gift of Dan Burke. It was derived by tetrad dissection from two parent strains that had been modified by one step gene replacements. The *pdr1::KanMX4* was constructed in BY4741 (*MAT*a *his3Δ1 leu2Δ0 met15Δ0 ura3Δ0*) and the *pdr3:KanMX4* was constructed in BY4742 (*MAT*α *his3Δ1 leu2Δ0 met15Δ0 ura3Δ0*). PCR-mediated one-step gene replacements, matings and tetrad dissections were performed as described[22]. Strains 9526-6-2-pRS416 and 9526-6-2/pRS416/PLP/HA were generated by transformation of 9526-6-2 with plasmids pRS416 and pRS416/PLP/HA, respectively, and were maintained on synthetic complete medium (SC) lacking uracil. For growth experiments and library screening, a single transformed colony was grown overnight and the cell number determined using a Coulter counter (Beckman Coulter Corporation). The cells were diluted to 5×10^5^ cells/ml in SC lacking uracil and containing 2% raffinose and 2% galactose. 95 µl of this culture was added to 5 µl of pre-plated test compounds in 96-well plates such that the final drug concentration was 50 µM and the final DMSO concentration was 1%. The Diversity Set library (National Cancer Institute Developmental Therapeutics Program) was used for the drug screen. It was provided as 10 mM stocks in 100% DMSO. OD_600_ readings were taken every 12 hours for 60 hours using a Thermomax microplate reader (Molecular Devices).

### Virus replication assays

Confluent cell monolayers of VeroE6 or MA104 cells were infected at an MOI of 3 with SARS-CoV/GFP. At 1 hour post infection, virus was removed and cells washed with PBS. Media was replaced with fresh MEM with either 1% DMSO or each drug diluted in 1% DMSO. At the identified time points, viral supernatant was removed and frozen at −80C until titering. SARS-CoV titers were determined by plaque assay on VeroE6 cells and Influenza viral titers were determined by TCID_50_ analysis as described [16]. For influenza virus replication, MDCK cells were infected at an MOI of 0.1 with influenza A/PR/8. Compounds were added at the indicated concentrations 1 hour post-infection at a final DMSO concentration of 1%. Cell supernatants were harvested after 48 hours and analyzed by hemagglutination assay. For SARS-CoV RNA analysis, Vero cells were treated with 50 um of NSC158362 at either 2 hours before, 0 hours before, 2 hours after or 6 hours after infection with SARS-CoV at an MOI of 3. At 12 hours past the zero time point, RNA was extracted and used for cDNA production. SARS-CoV leader containing mRNA, a sign of viral replication, was analyzed by PCR using forward primer CTCTTGTAGATCTGTTCTCTAAACGAAC and reverse primer TTACTGTACTAGCAAAGCAATATTGTCG.

### Luciferase reporter assay

To analyze the induction of IFNβ genes, a luciferase reporter assay was used in 293T cells. Two different inducers of Interferon were used for these experiments, RIG-I and Poly IC. For RIG-I transfection experiments, wells were co-transfected with a plasmid containing an IFNβ promoter fused to firefly luciferase (IFNβ/luciferase), a pCAGGS-PLP/HA plasmid [10], with or without pCAGGS-RIG-I. Two hours after transfection, each compound was added to the individual wells and incubated for 18 hours until analysis with the Luciferase Assay System (Promega) according to the manufacturer's protocol.

For Poly IC experiments, cells were co-transfected with the IFNβ/luciferase plasmid with or without the pCAGGS-PLP/HA plasmid [10]. At two hours post transfection each compound was added to the wells. At 18 hours post transfection, cells were transfected with 50 ug/ml of Poly IC (Sigma Aldrich) using Lipofectamine (Invitrogen). After an additional 6 hours incubation, cells were lysed and analyzed for luciferase induction using the Luciferase Assay System (Promega) according to the manufacturer's protocol.

### PLP activity assays

For protease activity experiments, PLP/HA was co transfected with or without the nsp2/3/GFP reporter described in [10]. At 4 hours post transfection drugs were added to wells at the indicated concentration. At 24 hours post transfection, the cells were lysed in RIPA buffer and visualized on SDS-PAGE gels with anti-GFP antibody (Sigma). For ubiquitination assays, a plasmid expressing Flag tagged ubiquitin was co-transfected with or without pCAGGS-PLP/HA and at 4 hours post transfection drugs were added at the indicated concentrations. At 24 hours post transfection, the cells were lysed in RIPA buffer and visualized on SDS-PAGE gels with anti-Flag antibody (Sigma).

### Human airway epithelial cells infections

Human nasal and tracheobronchial epithelial cells were obtained from airway specimens resected from patients undergoing elective surgery under UNC Institutional Review Board-approved protocols by the UNC Cystic Fibrosis Center Tissue Culture Core. Briefly, primary cells were expanded on plastic to generate passage 1 cells and plated at a density of 250,000 cells per well on permeable Transwell-Col (12-mm-diameter) supports. HAE cultures were generated by provision of an air-liquid interface for 4 to 6 weeks to form well-differentiated, polarized cultures that resemble in vivo pseudostratified mucociliary epithelium.

Apical virus inoculations were performed with 200 µl of virus stocks applied to the apical or basolateral surfaces of HAE. Following a 2 hour viral inoculation at 37°C, the inoculum was removed and drugs added to the apical surface in 5 ul volume and HAE were maintained with an air-liquid interface for the remainder of the experiment. At the indicated time points, the apical surface was washed with 200 ul PBS. This was then used for titering on VeroE6 cells for viral replication analysis.
